# Chromatin associated SETD3 negatively regulates VEGF expression

**DOI:** 10.1038/srep37115

**Published:** 2016-11-15

**Authors:** Ofir Cohn, Michal Feldman, Lital Weil, Margarita Kublanovsky, Dan Levy

**Affiliations:** 1 The Shraga Segal Department of Microbiology, Immunology and Genetics, Ben-Gurion University of the Negev, P.O.B. 653, Be’er-Sheva 84105, Israel; 2 National Institute for Biotechnology in the Negev, Ben-Gurion University of the Negev, P.O.B. 653, Be’er-Sheva 84105, Israel

## Abstract

SETD3 is a member of the protein lysine methyltransferase (PKMT) family, which catalyzes the addition of methyl group to lysine residues. Accumulating data suggest that PKMTs are involved in the regulation of a broad spectrum of biological processes by targeting histone and non-histone proteins. Using a proteomic approach, we have identified 172 new SETD3 interacting proteins. We show that SETD3 binds and methylates the transcription factor FoxM1, which has been previously shown to be associated with the regulation of VEGF expression. We further demonstrate that under hypoxic conditions SETD3 is down-regulated. Mechanistically, we find that under basal conditions, SETD3 and FoxM1 are enriched on the VEGF promoter. Dissociation of both SETD3 and FoxM1 from the VEGF promoter under hypoxia correlates with elevated expression of VEGF. Taken together, our data reveal a new SETD3-dependent methylation-based signaling pathway at chromatin that regulates VEGF expression under normoxic and hypoxic conditions.

SETD3 is a conserved histone H3 methyltransferase^1^. It is abundantly expressed in many tissues, including muscle, where it promotes myocyte differentiation by regulating the transcription of muscle-related genes^2^. Recent papers have also linked the expression of SETD3 to cancer progression. SETD3 was identified as novel biomarker for renal cell carcinoma (RCC)^3^: SETD3 expression was significantly higher in a set of RCC samples compared to normal renal tissues, and high expression of SETD3 was inversely correlated with disease-free survival^3^. In addition, it has been shown that a truncated version of SETD3 lacking the SET domain is highly expressed in lymphoma and that it displays oncogenic properties^1^. Overexpression of SETD3 in zebrafish was shown to lead to decreased cell viability and induction of apoptosis^4^. Thus, it seems that the specific role of SETD3 in cancer is still not clear. Furthermore, despite these emerging data suggesting that SETD3 regulates diverse biological processes, the protein network and the cellular signaling pathways in which SETD3 is involved remain largely unexplored.

In order to expand our understanding of the processes in which SETD3 participates, we have utilized the ProtoArray system^5^ to define the SETD3 interactome and have identified 172 new SETD3 interacting proteins. We further characterized the molecular cross talk between SETD3 and one of the identified proteins FoxM1 (Forkhead box protein M1). FoxM1 belongs to the Forkhead box superfamily of transcription factors that share a conserved DNA-binding domain^6^,^7^. Recent papers have shown that FoxM1 plays a key role in tumor development and progression^8^,^9^,^10^, regulation of cell cycle^11^,^12^ and control of DNA damage response^13^. Furthermore, FoxM1 was shown to play a central role in multiple oncogenic signaling pathways such as the phosphatidylinositol 3-kinase (PI3K)/Akt^14^, estrogen receptor (ER)^15^, and VEGF pathways^16^,^17^,^18^,^19^. Members of the VEGF family are master regulators of vascular development (angiogenesis) which is an important factor in the progression of metastasis and solid tumors growth^20^. Angiogenesis and activation of the VEGF signaling are tightly regulated under hypoxia conditions and therefore it is important to decipher the mechanisms which regulate VEGF expression under low oxygen level.

We demonstrate that SETD3 binds and methylates FoxM1 *in vitro* and in cells and that CRISPR/Cas9-mediated depletion of SETD3 resulted in increased VEGF transcription under hypoxia. We further show that under normoxic conditions, the interaction between SETD3 and FoxM1 takes place at chromatin and specifically at the VEFG promoter. However, under hypoxia conditions we observed decreased SETD3 and FoxM1 protein levels and a significantly weaker association between the two proteins. Moreover, under these conditions the occupancy of SETD3 and FoxM1 at the VEGF promoter was lost, leading to efficient transcription of VEGF. Together, our data suggest that the functional interplay between SETD3 and FoxM1 at chromatin regulates VEGF expression under low oxygen levels.

## Results

### Defining SETD3 interactome using the ProtoArray platform

**Figure 1:**
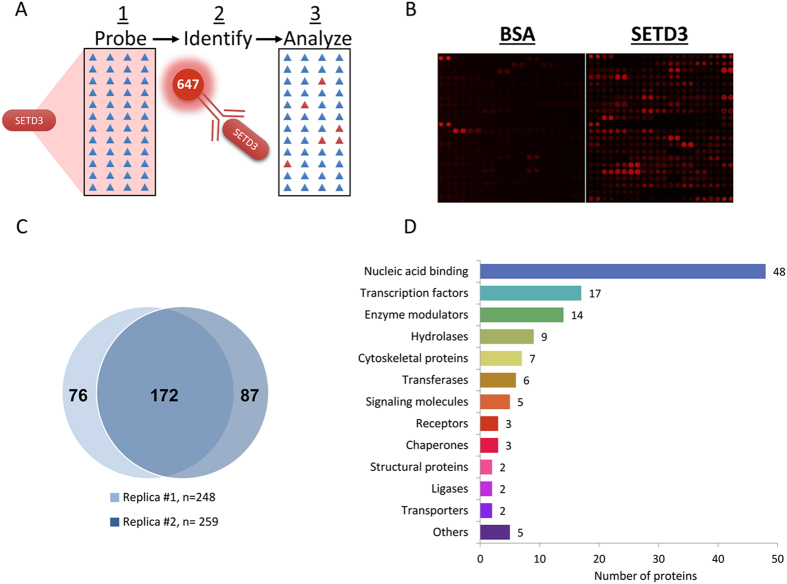
Identification of SETD3 interacting proteins with the ProtoArray system. (**A**) Schematic representation of the Proto-Array system (see Materials and Methods for more information). Briefly, arrays were probed with recombinant SETD3 (or BSA as a negative control) followed by identification of new interacting proteins using a specific SETD3 antibody. (**B**) Representative blocks of arrays that were probed with BSA (left) or SETD3 (right) recombinant proteins. (**C**) Venn diagram of the identified SETD3 interacting proteins identified in two independent experiments. (**D**) SETD3 interacting proteins were analyzed for gene ontology using PANTHER http://pantherdb.org/ 45. The number of proteins in each enriched term is indicated.

To identify new interacting proteins of SETD3, we performed a proteomic screen using the ProtoArray platform (Invitrogen). The ~9500 recombinant proteins printed on the array were probed with recombinant His-SETD3 followed by incubation with anti-SETD3 antibody (Fig. 1A). Representative blocks of the array that were probed with recombinant BSA (negative control) or His-SETD3 are shown in Fig. 1B. As illustrated in the Venn diagram of two independent experiments (Fig. 1C), the screen revealed 172 novel SETD3 interacting proteins with ~75% overlap between the two experiments. The new targets were divided into protein classes by gene ontology analysis (Fig. 1D). Of the 172 proteins, 65 were classified into “nucleic acid binding” (48 proteins) and “transcription factors” (17 proteins). A full list of the newly identified SETD3 interacting proteins is shown in Supplementary File 1.

### SETD3 binds and methylates FoxM1 *in vitro*

**Figure 2:**
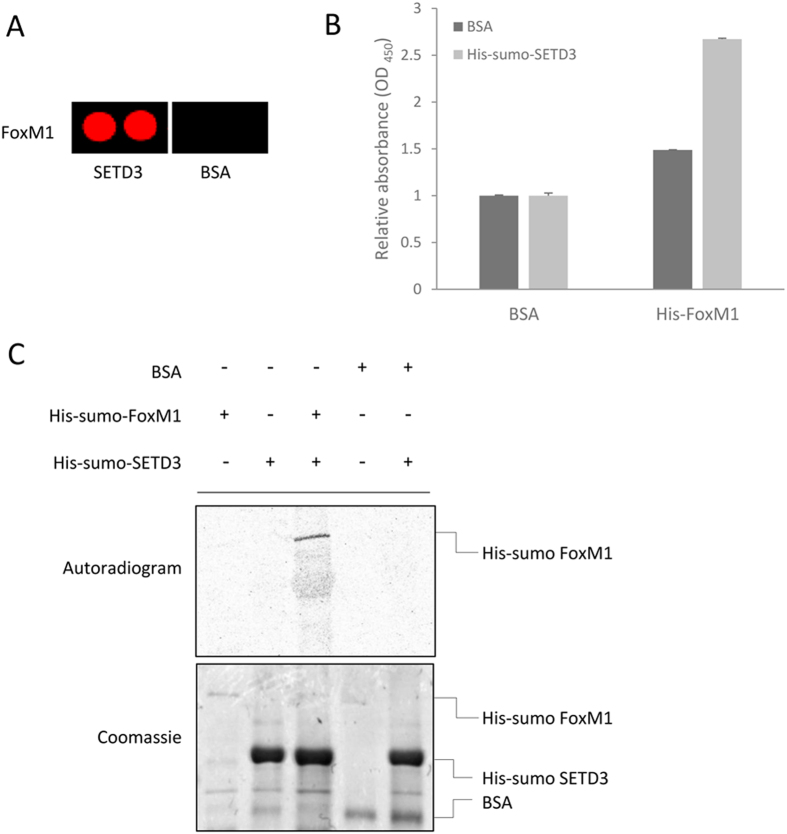
SETD3 binds and methylates FoxM1 *in vitro*. (**A**) FoxM1 Proto-Array signal from the arrays that were probed with SETD3 and BSA as indicated. (**B**) ELISA-based analysis of the interaction between recombinant His-sumo-SETD3 and His-sumo-FoxM1. Signal detection was achieved using primary anti-SETD3 normalized to BSA signal. (**C**) *In-vitro* methylation in the presence of ^3^H-labeled SAM with recombinant His-sumo-FoxM1, BSA (negative control) and His-sumo-SETD3. Coomassie stain of the recombinant proteins used in the reaction is shown below. Data are from at least two independent experiments (error bars, S.E.M).

As SETD3 was shown before to localize to the chromatin fraction^2^, we decided to concentrate on the transcription factor FoxM1, which is ranked highly on the list of SETD3 interacting proteins (Fig. 2A). To confirm direct interaction between FoxM1 and SETD3 we performed an ELISA experiment using recombinant purified proteins. As shown in Fig. 2B, we observed a strong interaction between the two proteins. Next, we examined if SETD3 methylates FoxM1. *In vitro* methylation assay with purified His-sumo-SETD3 and His-sumo-FoxM1 or His-FoxM1 showed that recombinant FoxM1 is methylated by SETD3 (Fig. 2C) and Supplementary Fig. S1A respectively). We then confirmed the specific methylation of FoxM1 by SETD3 with the ELISA 3XMBT affinity reagent method^21^,^22^ we have recently developed^23^ (Supplementary Fig. S1B). Collectively, these data led us to conclude that SETD3 binds and methylates FoxM1 *in vitro*.

### SETD3 interacts with and methylates FoxM1 in cells

**Figure 3:**
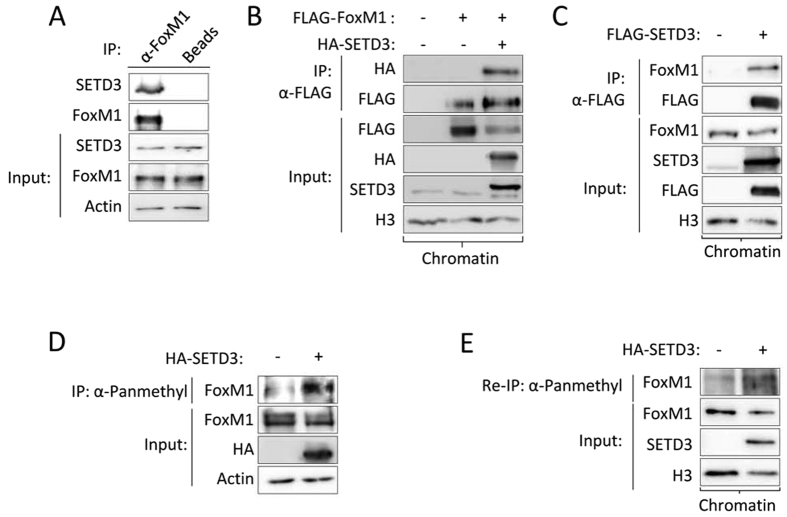
SETD3 interacts with and methylates FoxM1 in cells. (**A**) FoxM1 was immunoprecipitated from cells using an anti-FoxM1 antibody or control beads followed by western blot analysis with the indicated antibodies. Input is shown in the bottom panel. (**B**,**C**) HEK-293T cells were transfected with the indicated plasmids or with an empty plasmid. The chromatin fraction was then subjected to immunoprecipitation with FLAG M2 beads followed by western blot with the indicated antibodies. (**D**) Cells were transfected without or with HA-SETD3 and whole cell lysate was subjected to immunoprecipitation with a pan-methyl antibody followed by western blot with the indicated antibodies. (**E**) Chromatin isolated fraction of the transfected cells were subjected to immunoprecipitation with FoxM1 antibody followed by immunoprecipitation with pan-methyl antibody.

To determine if SETD3 binds FoxM1 in cells, we immunoprecipitated endogenous FoxM1 and found a specific interaction with endogenous SETD3 (Fig. 3A). Over-expression experiments revealed that the interaction between FLAG-FoxM1 and HA-SETD3 takes place at the chromatin fraction (Fig. 3B). Consistent with these results, a strong association at chromatin was observed between overexpressed SETD3 and endogenous FoxM1 (Fig. 3C). Immunoprecipitation of methylated proteins from HEK-293T cells using a pan-methyl antibody^5^ with or without over-expression of HA-SETD3 revealed a dramatic increase in the methylation of endogenous FoxM1 in the presence of SETD3 in whole cell extracts (Fig. 3D) and specifically at the chromatin fraction (Fig. 3E). Based on this set of experiments we concluded that SETD3 binds and methylates FoxM1 at chromatin.

### VEGF expression is negatively regulated by SETD3 and FoxM1

**Figure 4:**
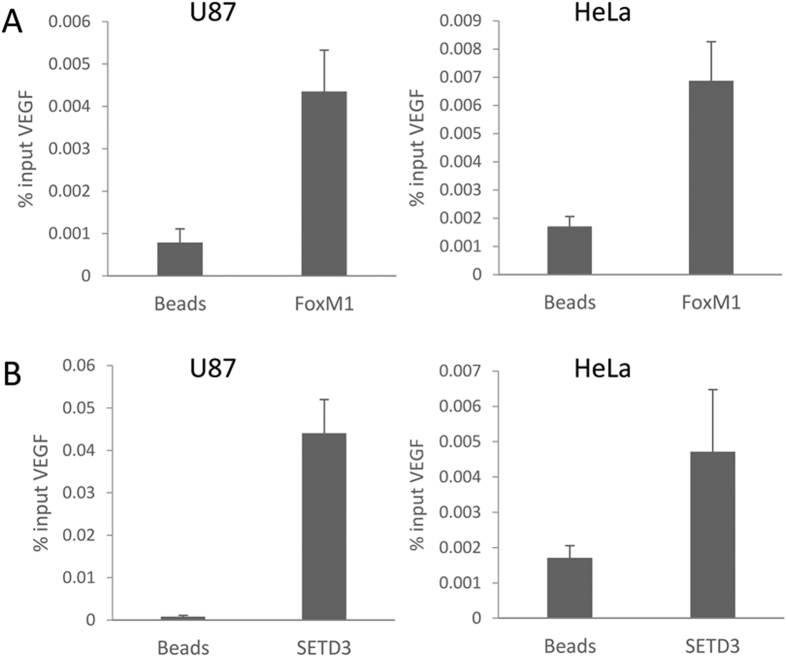
SETD3 and FoxM1 are enriched at the VEGF promoter. (**A**,**B**) ChIP-qPCR analysis of the occupancy of FoxM1 (A) and SETD3 (B) at the VEGF promoter in U87 and HeLa cells. Data are shown as % of input, from at least two independent experiments (error bars, S.E.M).

It was previously reported that FoxM1 has binding sites on the VEGF proximal promoter^7^, and we confirmed these findings in both U87 and HeLa cell lines (Fig. 4A). We therefore hypothesized that SETD3 is enriched on the VEGF promoter under basal conditions. Indeed, chromatin immunoprecipitation experiments revealed that SETD3 is enriched at the VEGF promoter under normoxic conditions (Fig. 4B). We thus hypothesized that SETD3 together with FoxM1 may regulate VEGF expression under hypoxia conditions.

**Figure 5:**
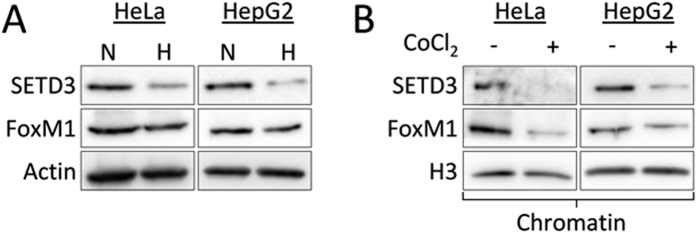
SETD3 is down-regulated under hypoxia. (**A**) HeLa and HepG2 cells were exposed to normoxic (N) or hypoxic (H, 1% O_2_) conditions followed by western blot with the indicated antibodies. (**B**) Cells were treated with 100 μM (HeLa) or 200 μM (HepG2) CoCl_2_ followed by western blot analysis of the chromatin fraction.

To study the effect of hypoxia on SETD3 expression levels we exposed HeLa and HepG2 cells to hypoxic conditions (1% O_2,_ for 24 h). A dramatic decrease in SETD3 protein level was observed in the cells that were exposed to hypoxia (Fig. 5A). Interestingly, we could also observe a slight decrease in FoxM1 protein level. As SETD3 binds FoxM1 at the chromatin, we next followed their expression at the chromatin fraction. In these experiments we have treated the cells with CoCl_2_. CoCl_2_ is a mimetic agent widely used *in vitro* to induce cellular responses mediated by hypoxia. CoCl_2_ mimics several aspects of the hypoxic response, such as increasing and stabilizing HIF-1α protein through inhibition of Prolyl Hydroxylases (PHDs) activity^24^. A significant reduction in the expression of both SETD3 and FoxM1 at chromatin in HeLA and HepG2 cells was observed (Fig. 5B).

**Figure 6:**
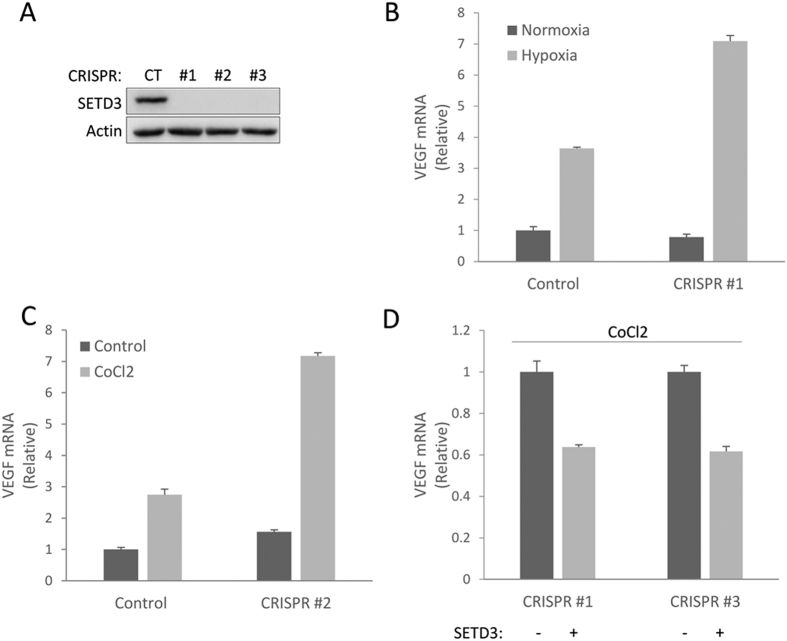
SETD3 negatively regulates VEGF expression. (**A**) Western blot analysis for control (CT) and 3 single SETD3 knock-out CRISPR clones of HeLa cells. (**B**,**C**) Real-time qPCR analysis of VEGF mRNA levels in control or SETD3 knock-out HeLa cells under normoxic or hypoxic conditions (**B**) or CoCl_2_ treatment (**C**). (**D**) Real-time qPCR analysis of VEGF mRNA levels with or without over-expression of SETD3 in two SETD3 knock-out HeLa cells treated with CoCl_2_. Data are from at least three experiments (error bars, S.E.M.).

To elucidate the role of SETD3 in the regulation of VEGF expression under hypoxia we generated SETD3 knock-out HeLa cells using the CRISPR/Cas9 system (Fig. 6A). We then measured VEGF mRNA levels using real-time qPCR in normal and in hypoxic conditions. The results revealed that VEGF expression was dramatically higher in the SETD3 knock-out cells after induction of hypoxia by 1% O_2_ (Fig. 6B) or CoCl_2_ (Fig. 6C) compared to control cells. In a rescue experiment, we treated SETD3 knockout cells with CoCl_2_ with or without overexpression of SETD3 and measured VEGF expression by qPCR (Fig. 6D). The results show a 40% reduction in VEGF expression when SETD3 was re-expressed in two independent SETD3 knock-out clones. Based on these results, we concluded that SETD3 negatively regulates VEGF expression under hypoxia.

**Figure 7:**
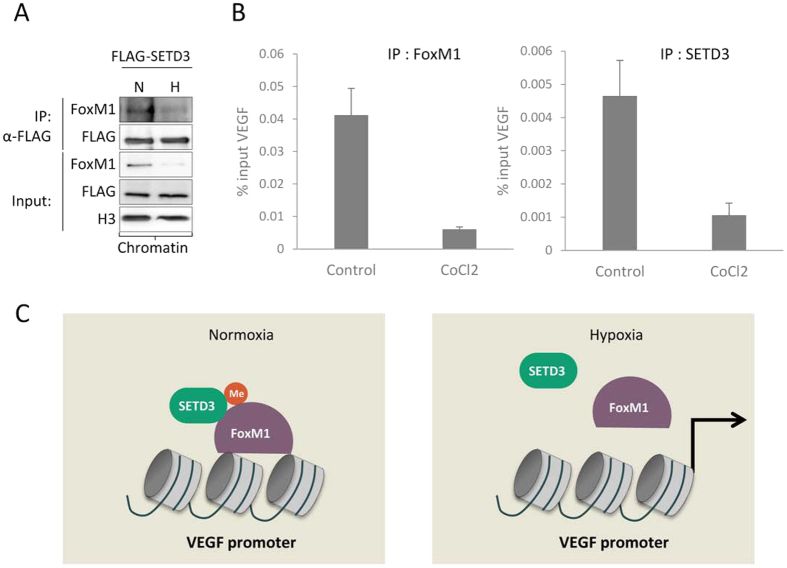
SETD3 dissociates from the VEGF promoter under hypoxic conditions. (**A**) HEK-293T cells transfected with FLAG-SETD3 were subjected to normoxic (N) or hypoxic (H) conditions. The chromatin fraction was then subjected to immunoprecipitation with FLAG M2 beads followed by western blot with the indicated antibodies. (**B**) Chromatin immunoprecipitation followed by real-time qPCR analysis of the occupancy of FoxM1 (left) or SETD3 (right) at the VEGF promoter in control and CoCl_2_-treated HeLa cells. Data are shown as % of input, from at least three independent experiments (error bars, S.E.M). (**C**) Suggested model for the interplay between SETD3 and FoxM1 at chromatin under normoxic and hypoxic conditions (see text).

We assumed that the binding of SETD3 to the VEGF promoter might determine the inhibitory effect of SETD3 on the expression of VEGF. To this end, we examined the interaction between SETD3 and FoxM1 at chromatin under normoxic and hypoxic conditions (Fig. 7A). We confirmed a strong interaction under normoxic conditions. However, a dramatic reduction in the association between SETD3 and FoxM1 at chromatin was observed under hypoxia. Consistent with these results, chromatin immunoprecipitation experiments revealed that the occupancy of SETD3 and FoxM1 at the VEGF promoter was lost under hypoxia (Fig. 7B). Collectively, our data may support a model (Fig. 7C) in which the methylation of FoxM1 by SETD3 at chromatin inhibits the activation of VEGF. Under hypoxic conditions, SETD3 and FoxM1 level is reduced, leading to a decreased interaction with FoxM1 and dissociation from the VEGF promoter and de-repression of VEGF transcription.

## Discussion

While a few human PKMTs have been characterized, the SETD3 interactome together with SETD3 cellular functions have not yet been elucidated. We have performed a proteome-wide screen to identify 172 novel SETD3 interacting proteins. In this study we concentrated on one of these interacting proteins, the transcription factor FoxM1, which participates in many cellular signaling pathways including sonic hedgehog, ERK, Raf/MEK/MAPK, and VEGF signaling. Through these pathways, FoxM1 is linked to numerous cellular and organismal processes, including cell cycle regulation, development, and tumorigenesis.

In this study we provide evidence for a new methylation signaling pathway at chromatin to regulate VEGF expression under normal and hypoxic conditions. We show that SETD3 specifically binds and methylates FoxM1 *in vitro* and in cells and that depletion of SETD3 from cells results in higher expression of VEGF under hypoxia. We show that SETD3 and FoxM1 co-occupy the VEGF promoter under normoxia. However, under low oxygen levels, SETD3 cellular levels and specifically at chromatin are down-regulated, leading to SETD3 and FoxM1 dissociation and efficient transcription of VEGF.

The cellular response to hypoxia is mediated in large part by members of the hypoxia inducible factor (HIF) family of proteins, which positively and negatively regulate the expression of multiple target genes. Liu and co-workers^25^ recently showed that HIF1 alpha and HIF2 alpha are methylated by SETD7 on lysines 32 and 29, respectively. This methylation inhibits the expression of HIF1/2 target genes. Kim *et al*.^26^ have shown a molecular interplay between SETD7 and the demethylase LSD1 to modulate HIF1 alpha transcriptional activity. FoxM1 methylation by SETD3 adds a new regulatory dimension to this complex process. As many non-histone proteins in the human proteome are known to be methylated^27^,^28^,^29^,^30^,^31^,^32^,^33^,^34^, we hypothesize that additional cellular factors that regulate the VEGF signaling under hypoxia may also be methylated by SETD3. Interestingly, two of the known VEGF receptors^35^, FLT1 (VEGFR1) and KDR (VEGFR2), were identified as SETD3 interacting proteins in our ProtoArray screen (Supplementary Fig. 1). Future work is required to further characterize these specific interactions. However, it clearly demonstrates that SETD3 might play a vital role in the VEGF signaling pathway.

The molecular mechanism by which FoxM1 modulates the expression of VEGF is still unclear.

Xia *et al*.^36^ have shown that HIF-1 binds directly to the FoxM1 promoter to upregulate FoxM1 expression under hypoxic conditions. In contrast, our results suggest that FoxM1 levels at chromatin are reduced under hypoxia. These differing results may be due to the different cellular compartments interrogated in the two different studies (whole cell lysates vs. insoluble chromatin fraction) or to differences in cell lines, antibodies and hypoxia conditions. Future studies will help define the directionality and molecular mechanism of FoxM1 regulation under basal and stimulated conditions.

FoxM1 is highly modified by post-translational modifications. For example, FoxM1 phosphorylation by PLK1 leads to FoxM1 activation and expression of key mitotic regulators^37^,^38^; SUMOylation inhibits FoxM1 activity and delays mitotic transitions^39^; and O-GlcNAc transferase (OGT)-dependent modification of FoxM1 regulates FoxM1’s transcriptional activity^40^. In a comprehensive proteomic screen using mass spectrometry, FoxM1 was found to be di-methylated at K278 and K282^41^. These two sites were not validated yet, however it will be interesting to determine whether these sites are targeted by SETD3.

In summary, our findings demonstrate a new methylation signaling pathway at chromatin which is mediated by SETD3 to regulate VEGF expression by FoxM1 under normoxic and hypoxic conditions. The formation of new blood vessels (angiogenesis) is a critical step for tumor progression and for solid tumor growth and hypoxia is one of the main physiological regulators of this process. While many years of research enabled us to better understand the molecular responses to hypoxia, the identification of new signaling pathways that mediate this process is useful for designing novel hypoxia related therapeutic intervention.

## Materials and Methods

### Cell culture, treatments and transfections

Human embryonic kidney (HEK-293T), hepatocellular carcinoma (HepG2), cervix carcinoma (HeLa) and glioblastoma (U-87MG) cells were cultured in Dulbecco’s modified Eagle medium (DMEM) supplemented with 10% fetal bovine serum (Gibco), 2 mg/ml L-glutamine (Sigma, G7513), penicillin-streptomycin (Sigma, P0781) and non-essential amino acids (Sigma, M7145) at 37 °C in a 5% CO_2_ incubator. For hypoxia experiments (1% O_2_) cells were incubated in serum-free medium for 24 h. CoCl_2_ (Sigma, C8661) was added at a final concentration of 100 μM (HeLa) or 200 μM (HepG2). For cell transfection, cells were plated in 6-well or 10-cm plates and transfected using Mirus transfection reagents (*Trans*IT®-LT1 for HEK-293T and HeLa cells, or *Trans*IT®-X2 for HepG2 cells) according to manufacturer’s instructions.

### Plasmids

Plasmids used for over-expression in cells were: pcDNA-HA-SETD3, pcDNA-FLAG-SETD3, pcDNA-HA-FoxM1, pcDNA-FLAG-FoxM1. For CRISPR/Cas9-mediated gene disruption, three different guide RNAs (gRNAs) for SETD3 were sub-cloned to the lentiCRISPR plasmid (Addgene, #49535). gRNAs sequences that target SETD3 are: #1-CGAGTAAAAACTCAGAAATC, #2-TACAGCAACTGTGTCACCAA,#3-GTATGTGCAGATCCGGACTC. Following transfection and puromycin selection, single clones were isolated and expanded. Plasmids used for expression and purification of recombinant proteins were: for the *in vitro* assays pGEX-6P1 SETD3 (kindly provided by Or Gozani’s lab) and pLX304 FoxM1 (kindly provided by Yair Bar’s lab) were sub-cloned into pETDuet and pET-SUMO vectors. pGEX-6p1-FLAG-MBT (provided by Or Gozani’s lab) was sub cloned into pETDUET vector.

### Recombinant protein expression and purification

*Escherichia coli* BL21, transformed with a plasmid encoding a protein of interest, were grown in LB media. Bacteria were harvested by centrifugation after IPTG induction and lysed by sonication on ice (25% amplitude, 1 min total, 10 sec on/off). The tagged fusion proteins were purified on His-Trap column using AKTA Pure protein purification system (GE).

### Western blots, immunoprecipitation and antibodies

Primary antibodies used were as follows: SETD3 (ab176582; Abcam), FoxM1 (GTX102170; GeneTex, HPA029974; Sigma), FLAG (F1804; Sigma), HA (05-904; Millipore), Actin (ab3280; Abcam), H3 (ab10799; Abcam), and pan-methyl (ab23366; Abcam). Secondary HRP-conjugated antibodies (goat anti-mouse and goat anti-rabbit) were from Jackson ImmunoResearch (115-035-062 and 111-035-144, respectively). Coomassie stain was purchased from Expendon (ISB1L).

Cells were lysed in RIPA lysis buffer (50 mM Tris–HCl pH 8, 150 mM NaCl, 1% Nonidet P-40, 0.5% deoxycholate, 0.1% SDS (v/v), 1 mM dithiothreitol (DTT) and Sigma protease inhibitor cocktail (P8340, diluted 1:100)). Lysates were incubated for 1 h at 4 °C with 10 μl protein A/G beads (Santa Cruz Biotechnology) as a pre-clear step. Pre-cleared lysates were incubated overnight at 4 °C with FoxM1 antibody (1 μg) or pan-methyl antibody (4 μg) conjugated to beads or beads only as a control. For over-expression experiments, cells were lysed as described above and incubated with FLAG-M2-affinity gel beads (A2220; Sigma). After incubation, beads were washed 4 times with lysis buffer, heated at 95 °C for 5 min in Laemmli sample buffer, and resolved by SDS-PAGE.

### Enzyme-linked immunosorbent assay (ELISA)

ELISA plates (Greiner 96 W) were incubated with 2 μg His–sumo-FoxM1 (or BSA as a control) for 1 h at room temperature. The plates were then washed with PBS supplemented with 0.1% Tween® 20 (PBST) and blocked with 3% BSA in PBST for 1 h. Following blocking, the plates were washed and covered with 0.5 μg His-sumo-SETD3 or BSA protein (negative control) for 1 h. Plates were then washed and incubated with primary antibody (anti-SETD3, 1:20,000 dilution) followed by incubation with secondary HRP-conjugated antibody (goat anti-rabbit, 1:2000 dilution). After addition of TMB (3,3′,5,5′-Tetramethylbenzidine) reagent and 1 N H_2_SO_4_, absorbance at 450 nm was detected using a Tecan Infinite M200 plate reader.

### *In vitro* methylation assay

Recombinant proteins were incubated overnight at 30 °C with 2 mCi H^3^-labeled S-adenosylmethionine (AdoMet; Perkin-Elmer) in methylation buffer (50 mM Tris–HCl pH 8, 10% glycerol (v/v), 20 mM KCl, 5 mM MgCl_2_ Reaction mixtures were resolved by SDS-PAGE, followed by autoradiography for the detection of methylation events and Coomassie staining to validate the presence of all proteins in the reaction. BSA served as a negative control. The MBT-ELISA approach to identify methylated proteins was performed as described in Cohen *et al*.^23^.

### ProtoArray

Human protein arrays (Version 5.0; ProtoArray) were stored at −20 °C until use. Arrays were blocked with blocking buffer (5 mM MgCl_2,_ 0.5 mM DTT, 0.05% Triton X-100, 5% glycerol, 1% BSA, 10% PBS) at room temperature for 1 h. Arrays were then washed with probing buffer and incubated for 1.5 h in a hybridization chamber (Agilent, Santa Clara, CA) in a reaction mixture containing 80 μg of purified His-SETD3 in probing buffer (0.1% Tween 20, 1% BSA, 10% PBS) in a total reaction volume of 950 μl. Arrays were washed four times with probing buffer while shaking at 50 rpm for 5 min at room temperature. Arrays were then incubated with SETD3 antibody for 1 hour at room temperature while shaking at 50 rpm. Arrays were washed four times with probing buffer while shaking at 50 rpm for 5 minutes at room temperature, followed by incubation for 1 h with Alexa Fluor 647 chicken anti-rabbit IgG (Invitrogen) diluted in probing buffer while shaking at 50 rpm. The arrays were washed four times as described followed by one wash with PBSx1 and then with DDW. The arrays then dried in a centrifuge at 300 rcf for 1 min. Arrays were scanned (Axon GenePix 4000B; Molecular Devices Inc.,Sunnyvale, CA, USA) and data were analyzed for each block using software alignment (Genepix Pro 7 software; Molecular Devices, Sunnyvale, CA) and gene array list (GAL) files supplied by the protein array manufacturer (Invitrogen).

### Statistical analysis

The data were analyzed by using the ProtoArray Prospector software V5.2 (Invitrogen) which collects all the signals from the proteins on the Array, calculates the mean value and the standard deviation. Then it calculates a Z-score which identifies all the upper probe signals compared to the total signals from the entire protein population. Proteins were defined as positive hits when Z-score >3.

### Chromatin immunoprecipitation (ChIP)

Chromatin immunoprecipitation (ChIP) was performed as described^42^. Briefly, formaldehyde cross-linked protein-DNA complexes were immunoprecipitated by overnight incubation with the indicated antibodies. Precipitated DNA fragments were extracted with Chelex 100 resin (Bio-Rad) as described^43^ and amplified by real-time qPCR with primers specific for the VEGF promoter. The sequences of the primers used were 5′-CCCCTTTCCAAAGCCCATTCC-3′ and 5′-CCTTCTCCCCGCTCCAACACCC-3′.

### Protein-protein chromatin immunoprecipitation

Protein–protein ChIP was modified from a published protocol^43^. After cross-linking, cells were harvested and washed twice with PBS and then lysed in 1 ml lysis buffer (20 mM Tris–HCl pH8, 85 mM KCl, 0.5% NP-40 and 1% protease inhibitor cocktail; 10 min on ice). Nuclear pellets were re-suspended in 200 μl nuclei lysis buffer (50 mM Tris–HCl pH 8, 10 mM EDTA, 1% SDS and 1% protease inhibitor cocktail; 10 min on ice) and then sonicated (Bioruptor, Diagenode) with high power settings for 3 cycles, 6 min each cycle (30 sec on/off). Samples were centrifuged (20 min, 13,000 rpm, 4 °C) and the soluble chromatin fraction was collected. The soluble chromatin was immunoprecipitated as described above, washed according to the published protocol, resolved by SDS-PAGE gel and analyzed by immunoblot.

### RNA extraction and quantitative RT-PCR

Total RNA was extracted with NucleoSpin RNA (Macherey-Nagel) according to the manufacturer’s instructions. Extracted RNA (200 ng) was reverse-transcribed into cDNA using iScript cDNA Synthesis Kit (Bio-Rad), according to the manufacturer’s instructions. Real-time qPCR was carried out using the UPL probe library system (Roche). All samples were amplified in triplicates in a 384-well plate LightCycler 480 System (Roche). Expression levels were normalized with GAPDH using the 2-DDCt method^44^. The real-time qPCR primers were the following: SETD3: forward, 5′-TGACAGACTCTACGCCATGAA-3′, reverse, 5′-GGCTCGGTAAAATGCAATG-3′; FoxM1: forward, 5′-ACTTTAAGCACATTGCCAAGC-3′, reverse, 5′-CGTGCAGGGAAAGGTTGT-3′; VEGF165: forward, 5′-GCAGCTTGAGTTAAACGAACG-3′, reverse 5′-GGTTCCCGAAACCCTGAG-3′; and GAPDH: forward, 5′-AGCCACATCGCTCAGACAC-3′, reverse 5′-AATACGACCAAATCCGTTGACT-3′.

## Additional Information

**How to cite this article**: Cohn, O. *et al*. Chromatin associated SETD3 negatively regulates VEGF expression. *Sci. Rep*. **6**, 37115; doi: 10.1038/srep37115 (2016).

**Publisher’s note:** Springer Nature remains neutral with regard to jurisdictional claims in published maps and institutional affiliations.

## Supplementary Material

Supplementary Information

Supplementary File S1
