# Supplementary material for: Chromatin associated SETD3 negatively regulates VEGF expression
Source: Sci Rep. 2016 Nov 15;6:37115. doi: 10.1038/srep37115 (PMC5109252; doi:10.1038/srep37115)
Supplement: Supplementary Information [file srep37115-s1.pdf]

Chromatin associated SETD3 negatively regulates VEGF expression

Ofir Cohn[1,2], Michal Feldman[1,2], Lital Weil[1,2], Margarita Kublanovsky[1,2] and Dan Levy[1,2#]

[1]The Shraga Segal Department of Microbiology, Immunology and Genetics and the [2]National Institute for Biotechnology in the Negev, Ben-Gurion University of the Negev, P.O.B. 653, Be'er-Sheva 84105, Israel

[#]Correspondence should be addressed to D.L: ledan@post.bgu.ac.il

Corresponding Author:

Dr. Dan Levy

Department of Microbiology, Immunology and Genetics

Ben-Gurion University of the Negev

Be'er Sheva 84105, Israel

e-mail: ledan@post.bgu.ac.il

Tel: +972-8-6477251

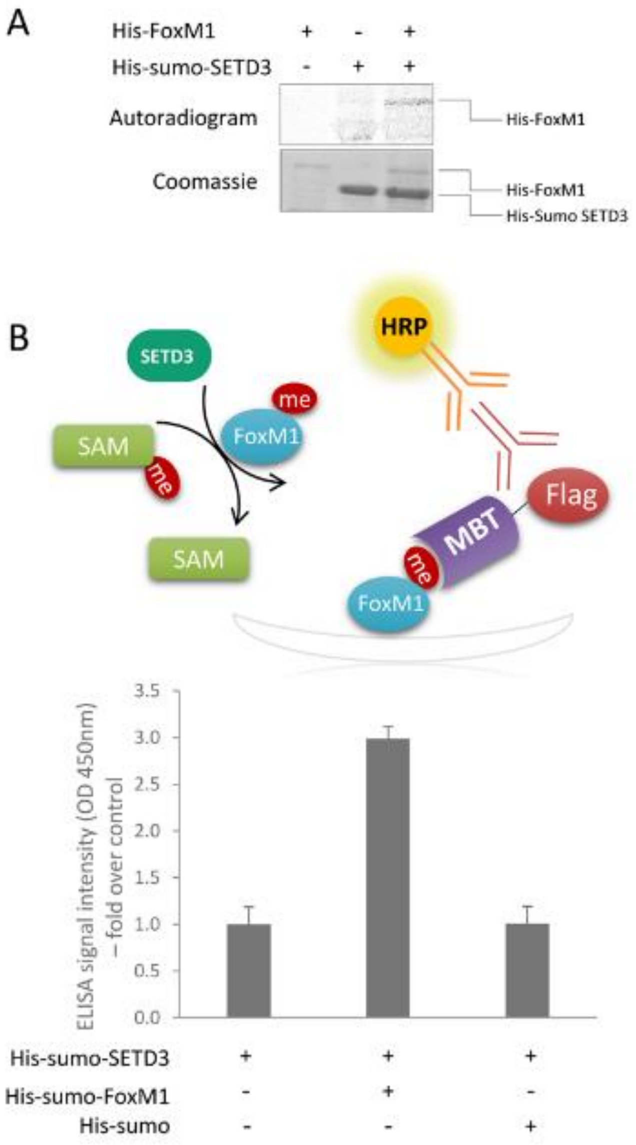

Figure S1: SETD3 methylates FoxM1 *in vitro* A. *In-vitro* methylation in the presence of ^{3}H-labeled SAM with recombinant His-FoxM1 and His-sumo-SETD3. Coomassie stain of the recombinant proteins used in the reaction is shown below. **B.** ELISA signal intensity (OD 450nm) of the indicated proteins that were subjected to in-vitro methylation reaction followed by detection with His-FLAG-MBT protein. Data are representative of three independent experiments (error bars, S.D.). Schematics of the ELISA approach for the identification of methylated proteins is shown above.
